# The Role of Genetic Pathways in the Development of Chemoradiation Resistance in Nasopharyngeal Carcinoma (NPC) Patients

**DOI:** 10.3390/genes12111835

**Published:** 2021-11-21

**Authors:** Norhafiza Mat Lazim, Che Ismail Che Lah, Wan Khairunnisa Wan Juhari, Sarina Sulong, Bin Alwi Zilfalil, Baharudin Abdullah

**Affiliations:** 1 Department of Otorhinolaryngology-Head & Neck Surgery, School of Medical Sciences, Health Campus, Universiti Sains Malaysia, Kubang Kerian 16150, Kelantan, Malaysia; cismailcl@usm.my (C.I.C.L.); baharudin@usm.my (B.A.); 2 Department of Microbiology and Parasitology, School of Medical Sciences, Health Campus, Universiti Sains Malaysia, Kubang Kerian 16150, Kelantan, Malaysia; wkhairunnisa@usm.my; 3 Malaysian Node of the Human Variome Project, School of Medical Sciences, Health Campus, Universiti Sains Malaysia, Kubang Kerian 16150, Kelantan, Malaysia; zilfalil@usm.my; 4 Human Genome Centre, School of Medical Sciences, Health Campus, Universiti Sains Malaysia, Kubang Kerian 16150, Kelantan, Malaysia; ssarina@usm.my

**Keywords:** nasopharyngeal carcinoma, chemoresistance, cancer stem cells, genetic alteration, fusion genes, locoregional recurrence, metastases, radiation resistance

## Abstract

Management of nasopharyngeal carcinoma (NPC) remains elusive despite new developments and advancement that has been made in the current management approaches. A patient’s survival and prognosis remain dismal especially for a late-stage disease. This is highly attribute to the chemoradiation resistance. Arrays of genes and molecular mechanisms underlie the development of chemoradiation resistance in NPC. Imperatively, unravelling the true pathogenesis of chemoradiation resistance is crucial as these significant proteins and genes can be modulated to produce an effective therapeutic target. It is pivotal to identify the chemoradiation resistance at the very beginning in order to combat the chemoradiation resistance efficiently. Intense research in the genetic ecosphere is critical, as the discovery and development of novel therapeutic targets can be used for screening, diagnosis, and treating the chemoradiation resistance aggressively. This will escalate the management trajectory of NPC patients. This article highlights the significance of genetic and molecular factors that play critical roles in the chemoradiation resistance and how these factors may be modified for next-generation targeted therapy products.

## 1. Introduction

Nasopharyngeal carcinoma (NPC) is a solid tumor composed of phenotypically and genotypically heterogeneous populations of neoplastic cells. NPC affects both the young and the elderly patients and has caused a significant burden on cancer statistics. NPC is a prevalent tumor in Southeast Asia, China, Taiwan, Hong Kong, the Middle East, and North Africa [1,2,3]. The geographical difference is attributed to different lifestyle and environmental risk factors. The incidence in males is slightly predominant with some familial inheritance [4]. The patient who had a positive family history of first-degree relatives has 4-fold increased NPC risks [5]. In the majority of cases, the patient presents with neck nodes and hearing complaints such as tinnitus and conductive hearing loss. Other presenting features include anosmia, blurring of vision, epistaxis, weight loss, and intracranial symptoms depending on the structure involvement. Young NPC patients have a predilection for more aggressive disease and carry poor prognoses [6,7]. Currently, there is a sharp increment in the young NPC cases observed in our practice. Of note, this tumor is known to be aggressive with local recurrence and distant metastases. The outcome of patients with distant metastases is very poor, with the median overall survival of 20 months [8].

Chemoradiation is the mainstay treatment for NPC. Generally, early-stage disease is highly radiosensitive and stage III–IV disease is treated with concurrent chemoradiation [9,10,11]. Older patients, despite having multiple comorbidities, may respond better to chemotherapy [7]. Platinum-based chemotherapy, cisplatin or carboplatin, is the typical first-line treatment for NPC. It also used for any recurrent or metastatic diseases and cisplatin is typically combined with fluorouracil [12,13,14,15]. Other chemotherapy agent includes Gemcitabine, a pyrimidine analog that inhibits DNA and has been used in combination with cisplatin in many trials of NPC [13,16,17]. The chemoradiation regimes are different between centers but generally is prescribed over 6 months. Collectively, some head and neck cancer patients might benefit from the combination of cisplatin and an immunotherapy such as EGFR inhibitor [18]. Gefitinib and erlotinib are another type of targeted therapy used for NPC [19]. Importantly, the EGFR signaling might play an important role in the resistance of head and neck cancer to cisplatin. Despite multimodality treatment, which has been in practice for decades for treating head and neck cancers, the desired treatment outcomes remain suboptimal.

Drug resistance, either intrinsic or acquired, is one of the major significant challenges in the recent treatment armamentarium of NPC. In practice, the majority of NPC patients present with late-stage disease and are unresponsive to chemoradiation [20,21]. This may be attributed to the chemoradiation resistance [22,23]. Tumors recurring within 1 year are considered as radioresistant [23]. Resistance to anticancer drugs arises from a wide variety of factors, such as genetic mutations, epigenetic changes, drug efflux, and various other cellular and molecular mechanisms. Multiple studies have shown that chemoradioresistance-associated molecules, such as mRNAs, microRNAs, and proteins, influence radio resistance by regulating radioresistance-associated processes, including DNA repair capacity, apoptosis, cell cycle arrest, and protective autophagy [24]. MiRNAs, for example, have a role in cytochemical or radiation resistance [25,26,27,28,29].

## 2. Chemoradiation Resistance in NPC

Although NPC patients are sensitive to chemotherapy during the initial treatment, they develop acquired resistance shortly after, leading to treatment failure. Many crucial factors contribute to the development of radioresistance in NPC. The molecular mechanisms that have been proven to contribute to the chemoresistance include drug efflux, DNA damage and repair, epithelial-mesenchymal and autophagy, exosomes, and Epstein Barr virus (EBV) [30,31]. Evasion of apoptosis by cancer cells is the significant cause of chemoresistance. Additionally, Cancer Stem cells (CSCs) have been shown to be increasingly related to the chemoresistance and are associated with multiple interactions with specific markers, extracellular matrix, proteases, cytokines, and fibroblast.

Significant recent literature highlights the roles of microRNAs (miRNAs) in the pathogenesis of drug resistance. NPC carcinogenesis and development of chemoradiation resistance have all been linked to dysregulated microRNAs [32]. By altering the proliferation, apoptosis, invasion, and metastasis of NPC cells, these miRNAs can influence the genesis and progression of NPC. MiRNAs can affect the transmission of related signaling pathways by regulating the expression of tumor suppressor genes and/or oncogenes, and thus participate in radiotherapy resistance in NPC [32]. The EBV is a significant risk factor for the development of NPC. Critically, EBV produces abundant miRNAs, which contribute to carcinogenesis and chemoradiation resistance by expression of specific proteins such as Mir BART [31].

Recent research focus has shifted to the development of new drugs that can enhance patient prognosis and survival with fewer related complications. By understanding the true mechanism of chemoradiation resistance, a potent therapeutic target can be developed for clinical application in the head and neck oncology management armamentarium. Thus, clarifying the molecular mechanisms of chemoradiation resistance in NPC is important for developing a personalized and precise therapeutic approach. In this review, we will discuss the key genes that are involved in chemoradiation resistance in NPC and their specific mechanisms. In addition, a brief description of the pathways of implicated genes or proteins and their potential applications in fighting against drug resistance and improving anticancer efficacy will be highlighted.

## 3. Key Genes and Molecular Mechanisms of Chemoradiation Resistance in NPC

There are multiple critical mechanisms that underlie the chemoradiation resistance in NPC. This involves arrays of genes, proteins, and peptide products, which interact in a delicate system that play critical roles in modulation of the chemoradiation effects [32]. The comprehensive studies of genes and proteins are based on the fact that multiple genes can interact with signaling pathways to regulate radiosensitivity, and selected gene fusion can induce chemoradiation resistance. The activation of oncogenes or the loss of tumor suppressors plays important roles in tumor initiation and progression, which can also regulate the EMT process, CSC formation, and radiosensitivity of NPCs. All of these factors act in a cohesive ecosystem in inducing chemoradiation resistance in NPC.

### 3.1. Epithelial–Mesenchymal Transition (EMT)

Epithelial–mesenchymal transition (EMT) is a process by which cells change their original epithelial morphology, i.e., the epithelial cells transform into mesenchymal cells. The EMT has significant roles in cancer progression and metastases [33]. EMT allows solid tumors to become more malignant and invasive and have more metastatic potential. Recently, compelling evidence indicated that EMT transition in tumor cells also contributes to other malignant behaviors, such as chemoresistance and radioresistance [31].

Many facets of critical tumor growth are aided by EMT functions. Some chemo-resistant NPC cells have EMT features, implying that EMT is a type of chemo-resistance maintenance mechanism in NPCs. In NPC, Zhang et al. discovered a link between EMT and cisplatin resistance. They discovered that cisplatin-resistant NPC cells had more EMT characteristics, such as decreased E-cadherin expression and increased vimentin, fibronectin, and matrix metalloproteinase expression [34].

### 3.2. Carnitine Palmitoyl Transferase 1A (CPT1A)

Carnitine Palmitoyl Transferase 1A is a rate-limiting enzyme for mitochondrial fatty acid transportation, which plays a critical role in increasing fatty acid oxidation required for the cellular fuel demands. High expression of CPT1A promotes radiation resistance in NPC cells and contributes to poor overall survival of NPC patients following radiation therapy [35]. Disruption of CPT1A decreases radiation resistance by activating mitochondrial apoptosis both in vitro and in vivo [36]. Current research highlights the true mechanisms of CPT1A involvement in lipid reprogramming and this may serve as a new platform for the development of molecular-targeted treatment in order to improve the therapeutic effects of radiation in NPC.

The reprogramming of lipid metabolism is a relatively emerging characteristic of cancer. Increased lipid uptake, storage, and lipogenesis are all linked to fast tumor growth in a variety of malignancies. The sterol regulatory element-binding proteins (SREBPs) are a family of membrane-bound transcription factors found in the endoplasmic reticulum that play a key role in lipid metabolic control [37]. SREBPs are substantially upregulated in many malignancies and promote tumor growth, according to recent research. Importantly, SREBPs are interesting therapeutic targets since inhibiting them genetically or with pharmacological treatments greatly reduces tumor development and triggers cancer cell death. However, directly blocking SREBPs, on the other hand, is difficult because transcription factors are notoriously difficult to target with drugs. Inhibiting SREBP translocation from the ER to the Golgi is a more promising strategy. Fatostatin, betulin, and PF-429242 have been proven in pre-clinical tests to suppress SREBP activation and have promising anti-tumor properties [37].

### 3.3. RARS-MAD1L1 Fusion Gene

The chromosome translocations and relevant gene fusions may contribute to tumor progression, but also the fusion protein produced by a fusion gene can be oncogenic [38]. An example of this fusion gene includes *RARS-MAD1L1*, which can be found in primary NPC and induces cellular proliferation and stem cell properties in NPC [39]. The human far upstream element (FUSE) binding protein 1 was activated by *RARS-MAD1L1* and was associated with poor survival in esophageal carcinoma [40]. Other examples include the *FGFR3-TACC3* fusion gene, which promotes cell proliferation, colony formation, and transforming potential via activating the ERK and AKT signaling pathways.

### 3.4. Base Excision Repair (BER) Pathway

The base excision repair (BER) pathway has been identified as a predictor of therapeutic response, prognostic factor, and therapeutic target in a variety of cancers. The BER pathway is made up of a variety of glycosylases, endonucleases, polymerases, and ligases that work together to repair damaged DNA bases. Cancer cells take advantage of BER’s ability to repair DNA damage in order to resist DNA-damaging chemotherapies and radiotherapy. BER proteins have thus been identified as potential therapeutic targets and chemoresistance factors in a variety of cancers [41].

Base excision repair (BER) pathway is the main way to repair the radiation-induced DNA single-strand break, including the apurinic/apyrimidinic (AP) site break and DNA base injury. In a study by Wang et al., three genes, *XRCC1, OGG1,* and *APEX1,* in the BER pathway were studied in 174 NPC patients who were treated with chemoradiation with five potentially functional single nucleotide polymorphisms (SNPs). They reported that that *XRCC1* and *OGG1* had a significant impact on primary tumor efficacy at the end of radiotherapy [42], which may have effects on the chemoradiation resistance.

### 3.5. Bone Marrow Stromal Cell Antigen 2 (BST2) and NF-κB Pathway

BST2 was identified as a platinum-resistant factor in NPC patients. The upregulation of BST2 resulted in platinum resistance by activating the NF-κB pathway to promote the expression of anti-apoptotic proteins. It has been shown that BST2 upregulation was associated with poor survival in patients with locally advanced NPC [43]. NF-κB deregulation, either via somatic genetic events or LMP1 overexpression, is another core feature of NPC [44,45]. Abnormal NF-κB signaling and genetic mutations in NF-κB signaling-associated factors impact the tumorigenicity, proliferation, chemoresistance, and radioresistance of multiple kinds of cancers including NPC [38]. Normally, a variety of stimuli can initiate NF-κB signaling, such as cytokines, growth factors, reactive oxygen species, and ionizing radiation [46].

### 3.6. Endoplasmic Reticulum (ER) Stress Pathway

ER stress may be activated by tumor microenvironment factors such as hypoxia, pH changes, and oxidative stress induced by chemoradiation, which results in cancer and metastases. Functional polymorphisms in ER stress pathway-related genes, especially SNPs in the coding regions, may affect the expression and activity of proteins, which might be predictive factors for chemoradiotherapy efficacy and may account for the inter-patient response heterogeneity and chemoradiation resistance [47].

### 3.7. Wnt/β-Catenin Pathway

Activation of Wnt-β catenin pathway has been associated with tumorigenesis and cancer progression in various types of cancers. Wnt/β-catenin signaling pathway maintains the capability for self-renewal and differentiation and induces radioresistance in several human cancers including NPC [48,49]. Elucidation of the molecular details of the oncogenic activation of Wnt/β-catenin signaling may, therefore, lead to more effective treatments for patients with NPC [49].

β-catenin may play a role in the migration and proliferation of NPC cells. The SNPs were correlated with grade 3 radiation-induced toxic reactions in NPC patients [50]. This shows that the accumulation of β-catenin could promote the proliferation of NPC cells and weaken the efficacy of radiation.

### 3.8. Long Noncoding RNAs (lncRNAs)

Long noncoding RNAs (lncRNAs) are generally primary non-protein-coding sequences greater than 200 nucleotides in length. They can interact with DNA, RNA, and proteins [51]. They are a novel class of mRNA-like transcripts that have been shown to be involved in the development and progression of multiple cancers. The lncRNAs regulate many hallmarks of cancer, such as the malignant phenotype, by regulation of the epithelial-to-mesenchymal transition process (EMT), and influence cancer invasion and metastasis [52].

They also underlie the chemoradioresistance in NPC. Furthermore, multiple lncRNAs are abnormally expressed in cancer cells and have been linked to the establishment of cancer cells’ radioresistant character. Increasing evidence suggests that lncRNAs affect the transcription of genes involved in the DNA damage response via a variety of regulatory mechanisms [51]. Studies analyzing large clinical cancer samples demonstrated that certain lncRNAs serve as valuable prognostic biomarkers [53,54].

### 3.9. Tyrosine-Protein Phosphatase (SHP-1)—CK2 Inhibitor

Protein Kinase (CK2) is an important kinase expressed in most eukaryotes. The CK2 complex is a tetramer composed of two catalytic subunits, which play a vital role in cell growth, proliferation, differentiation, and apoptosis and are considered to be potential targets for regulation of processes such as cell cycle distribution, apoptosis, and DNA damage repair. SHP-1 was highly expressed in NPC tissues in contrast to normal nasopharyngeal mucosa and was associated with local recurrence and metastasis after radiotherapy in NPC patients [55], which could point to its roles in chemoradiation resistance.

### 3.10. C-Jun Gene

C-Jun is a major constituent of activating protein transcription factor that transduces multiple mitogen growth signals [56]. Overexpression of c-Jun/AP-1 has been associated with tumor invasion, metastasis, and prognosis in many human cancers [56,57]. Previous studies have shown that the expression of cyclin is positively correlated with radiation resistance. The expression of c-Jun was significantly upregulated and may be associated with the radio-resistance of NPC [58]. C-Jun may play an essential role in radio-resistance through the EGFR pathway or AP-1. The finding implies that the overexpression of c-Jun may serve as a potential target to enhance the radiation sensitivity for NPC therapy.

### 3.11. MicroRNA (miRNA)

MicroRNAs are short non-coding RNAs, typically 21–23 nucleotides long, that function in post-transcriptional gene regulation typically through translation inhibition and mRNA degradation [59]. Specific individual miRNAs may be informative of changes with respect to cellular growth, proliferation, metastases, and apoptosis and for use in diagnostic or prognostic assessments. Other groups of miRNAs might guide the course of treatment by reflecting NPC resistance to radiotherapy or chemotherapy [60].

NPC has a high degree of expression of EBV-encoded microRNAs, particularly BART miRNAs, which are encoded from the viral genome’s BamHI-A region. According to growing data, ebv-mir-BARTs play a vital role in host cell survival, immunological evasion, cell proliferation, cell apoptosis, and cancer metabolism, boosting NPC formation. In NPC cell lines and primary tissues, ebv-mir-BART21 and ebv-mir-BART22 are substantially expressed [61]. Importantly, BART22 can suppress the host immune response by downregulating LMP2A at the translational level, allowing NPC cancer cells to survive.

The importance of miRNAs in chemotherapy response has been demonstrated in NPC. For instance, miR-3188 has been found to inhibit cell growth and resistance to fluorouracil by directly targeting the mechanistic target of rapamycin kinase gene, *MTOR*, and regulating the cell cycle. Another study reported that the metastasis suppressor miR-29c can also increase NPC cells’ sensitivity to both radiotherapy and cisplatin-based chemotherapy [62]. Tian et al. described the molecular mechanisms of miRNA involved in the radiotherapy resistance of nasopharyngeal carcinoma by affecting apoptosis, DNA damage repair, and cell cycle progression of nasopharyngeal carcinoma cells [20].

### 3.12. Cancer Stem Cells (CSCs)

Cancer stem cells (CSCs) are elucidated as cells that can perpetuate themselves via autorestoration. These cells are highly resistant to current therapeutic approaches and are the main reason for cancer recurrence. CSCs have been observed to drive tumor initiation and tumor chemoradioresistance [20,51,63,64]. Emerging evidence strongly supports that cancer stem cells may contribute to NPC’s resistance to chemoradiation [65,66]. CSCs have stronger radioresistance and particular molecular features that protect them from radiation-induced damage as compared to tumor cells’ mass [38]. This promotes the chemoradiation resistance in NPC patients.

Imperatively, CSCs’ subpopulations are able to express various markers that have different phenotypic and functional characteristics, even within the same tumor [67]. The propagation of CSCs to maintain the tumor initiation ability refers to the self-renewal of CSCs [68,69]. The CSCs’ resistance to chemoradiation is the source of local recurrence and metastasis [70]. CSCs are highly tumorigenic compared to the other cancer cells and are largely responsible for numerous biological characteristics of cancer [71,72]. CSCs have been found to have a strong DNA repair capacity in a variety of tumor types. Protection from oxidative stress by ROS scavenging, activation of anti-apoptotic pathways, and protection by microenvironmental niche 3 are among the mechanisms of CSC radioresistance.

Table 1 summarizes the list of key genes or biological pathways with the molecular mechanisms in NPC.

## 4. How to Overcome Tumor and Chemoradiation Resistance in NPC

Management of NPC presents specific challenges because of the complexity of the anatomical structures, the need for a multidisciplinary team, and the lifelong follow-up required for the patients. It goes without saying that early treatment saves a life. The genetic abnormalities in NPC drive the cell towards unrestricted growth by two general classes of genes, proto-oncogenes and tumor suppressor genes.

The mechanisms of chemoradiation resistance in NPC cells are influenced by several factors, and many approaches on how to overcome the course of chemoradiation resistance have been investigated. Among the major challenges of chemoradiation resistance is the evasion of apoptosis; inhibition of associated genes and signaling pathways have been extensively studied. The *Livin* gene that encodes anti-apoptotic protein was found to be involved in the overgrowth of cancer cells [81]. Inhibition of the *livin* gene can prevent the development of radioresistance in the NPC cells [82]. It has also been found that high expression of non-homologous end joining (NHEJ) machinery contributes to the resistance to conventional therapies [83].

Carcinogenesis may result from chromosomal aberrations caused by unrepaired DNA double-strand breaks (DSBs), and the NHEJ pathway was known as the main DSB repair pathway. DSBs are the most critical factor in stabilizing the genome maintenance systems as they can induce apoptosis if unrepaired and can cause chromosomal aberrations if mis-repaired, which will consequently contribute to cancer formation [84]. One of the reasons for resistance to chemoradiation itself is caused by the activation of DSB repair genes, which could be an important approach for the treatment of cancer [78]. In addition, high mobility group protein B1 (HMGB1) encodes by the *HMGB1* gene, is involved in several cellular processes including transcriptional regulation and DNA repair, and plays a role in the efficiency of NHEJ pathway [79]. Through NHEJ, *HMBG1* promotes DNA ligation in vitro and repairs DSB with an incompatible end that is normally caused by ionizing radiation. Due to its role in NHEJ machinery, a study by Zhu et al. found that inhibiting HMGB1 can overcome chemoradiation resistance in NPC.

A previous study of the retinoic acid-inducible gene I (RIG-I)-like receptor (RLR) family, which includes RIG-I, reported that RIG-I activation by its ligand could induce apoptosis in several tumor cells [85]. The effect of RIG-I was also elucidated on paclitaxel resistance in NPC cells, whereby overexpression of RIG-I increased the sensitivity of NPC cells to paclitaxel and suppressed cancer progression [86]. Reduced expression of JAK, IFN, and ER stress response signaling pathway factors was further observed in RIG-I knockdown cells, which suggested that RIG-I could be a target receptor in regulating the IFN/JAK2 and ER stress response to induce apoptosis and, consequently, inhibit chemoradiation resistance in NPC [86].

One reason for the failures of chemotherapy is multidrug resistance (MDR) [80]. Multidrug resistance is defined as the resistance to multiple chemotherapeutic drugs with different structures and functional activities [73,87]. The basic underlying MDR mechanism is described as follows: (1) increased drug efflux, (2) decreased drug influx, (3) increased drug metabolism, (4) increased DNA repair, and (5) decreased apoptosis [88]. The ATP-binding cassette (ABC) transporter proteins, such as P-glycoprotein (P-gp/MDR1), multidrug resistance-associated protein 1 (MRP-1), and breast cancer resistance protein (BCRP) play major roles in drug efflux-related mechanisms [89]. Due to the nature of the tumors that are multiclonal and heterogeneous, combinational therapies are highly preferred. Using two or more drugs prevents the development of new cancer mutations by targeting multiple driver genes concurrently. The simultaneous multi-targeting enhances the efficacy of anticancer therapies as the driver genes’ protein responsible for drug resistance pathways is destroyed. Inhibiting the energy supply of tumor cells is the other strategy of thwarting resistance development as the tumor needs the energy to grow and proliferate [90,91].

Drug resistance in tumor is acquired via cellular and non-cellular mechanisms. Overexpression of the plasma membrane P-glycoprotein (P-gp), responsible for the cellular mechanism, is capable of repelling drugs from the cell, causing decreased sensitivity and intracellular accumulation of drugs. P-glycoprotein binds to drugs, forming transmembrane channels, pumping the compounds out of the cells [92]. This can be circumvented by using encapsulated anticancer drugs with P-gp inhibitors delivered as nanoparticles to prevent P-gp-mediated MDR [92]. Poor vascularity and the acidic environment surrounding the cancerous cells accounted for the non-cellular resistance. A drug will have difficulty eradicating the cancerous cells as an insufficient amount is delivered for it to be effective due to a poor blood supply. In addition, the acidic environment inactivates the drug compounds, preventing their diffusion across the cellular membrane.

Compounds that can reverse the chemo drug resistance is identified as chemosensitizers or drug resistance modulators. They are classified as first-, second-, and third-generation agents. First-generation modulators are drugs that were initially developed for other purposes but incidentally found to be useful for enhancing tumor response towards chemotherapy. Some of these are tamoxifen, erythromycin, calcium channel blockers, antimalarials, and immunosuppressant agents [93]. The second-generation modulators are designed from the modification made out of the first-generation modulators to improve their efficacy and minimize their side effects [94]. Third-generation modulators are designed with better efficacy and fewer adverse effects due to a lesser effect on cytochrome p450, which accounts for most of the side effects observed. These include tariquidar (anthranilamide derivative), biricodar (pipecolinate derivative), Annamycin (anthracycline derivative), mitotane (2,4-dichloro-diphenyldichloroethane derivative), zosuquidar (dibenzosuberane derivative), and laniquidar (benzazepine derivative) [95].

The root extract of *Stemona curtisii* has improved the response of cervical cancer patients toward vinblastine, paclitaxel, and colchicines [96]. Another new class of chemosensitizers is flavonoids. Flavonoid, a natural compound widely present in foods and herbal products [97], was found to inhibit a protein responsible for breast cancer resistance. It acts on both the ATP binding site and the P-gp [98]. Various flavonoids such as biochanin A, diadzein, fisetin, morin, naringenin, quercetin, and silymarin have been investigated for their effect on P-gp function in tumor cells [99]. Biochanin A and silymarin have shown the most effective activity in potentiating the cytotoxicity effect of the drug and appear to be potent and safe P-gp inhibitors. Hence, the simultaneous administration of the flavonoids is expected to augment the efficacy of chemotherapeutic agents.

An ideal drug delivery technology is an ability of a drug system to stay in the blood flow and allow a continuous drug release at a particular target site. To ensure this system will work efficiently as a cancer therapeutic, it must be able to control and sustain the release of a specific drug to allow sufficient accumulation in the tumor cells [100]. There are two general classes available, the lipid-based and polymer-based systems. Lipid-based systems have the advantage of being easy to prepare, with higher bioavailability to free the drug, and capable of solubilizing hydrophobic drugs. The polymeric systems possess the same advantages as lipid-based systems but additionally are immunogenic. Chemotherapy drugs such as doxorubicin, which is categorized as a stealth liposome, have been used successfully in patients with head and neck cancers and other cancers [101].

Nanotechnologically targeted cancer chemotherapy offers a solution to overcome drug resistance tumor phenotype. It can be used to deliver specific drugs to a targeted area without harmful effects to the surrounding organs or tissue [24,102]. Among the known nanotechnologies being developed for pharmaceutical use are polymer-based nanoparticles, lipid-based nanoparticles, self-assembled nanostructures, and dendrimer-based nanostructures. These include nanocapsules, nanoparticles, nanorods, nanofibers, nanocrystals, nanotubes, stealth nanoparticles, liposomes, stealth liposomes, pH-sensitive liposomes, temperature-sensitive liposomes, etc. They possess a small particle size and narrow size distribution, with surface features for target-specific localization, protective insulation of drug molecules, opportunity to develop nanocarriers that respond to physiological stimuli, feasibility for delivery of multiple therapeutic agents in a single formulation, combination of imaging and drug therapy to monitor effects in real time, and the opportunity to combine drugs with energy (heat, light, and sound) delivery for synergistic therapeutic effects [103,104,105]. Multiple genes can interact with signaling pathways to regulate radiosensitivity, and their encoding proteins can act as potential biomarkers for predicting the reaction for radiotherapy. Epigenetics can regulate radioresistance of NPC as well. A number of miRNAs and lncRNAs may not only act as either oncogenes or tumor suppressors, regulating cell proliferation, migration, invasion, and metastasis, but also may be involved in the EMT process, CSC formation/phenotype, and radioresistance of NPCs, revealing predicting roles.

The advantage of miRNA-based treatment approaches is that miRNAs can concurrently target multiple effectors of pathways involved in tumor cell differentiation and proliferation. Thus, miRNA-based cancer treatments, alone or combined with standard chemotherapy and/or radiotherapy, hold promise to improve treatment response and cure rates [24]. Future scientific research on radioresistance of NPC can be tailored in several aspects of several focusing areas. The first aspect is to explore relevant methods that could sensitize CSCs to radiation. Since the co-existence of EMT and CSCs may be the common mechanisms for NPC radioresistance, blocking approaches or inhibiting the EMT process could be of potential research.

In addition, radioresistance is caused by several signaling pathways that interact with many key associated genes. By discovering more details on the related signaling pathways and the associated key genes, including biomarkers, we may elucidate other factors that influence radioresistance and improve patient prognosis [38]. Dentification of new miRNA or lncRNA should be taken into consideration for future research as NPC was reported to have the highest studies of association between miRNA and radioresistance [106]. A better knowledge of the mechanisms that contribute to radioresistance in NPC would allow for the development of treatments that induce tumor remission while reducing the amount of radiation required to achieve tumor control, thus improving the prognosis and quality of life of patients.

## 5. Conclusions

A major proportion of NPC patients present with recurrent and metastatic diseases despite a combined treatment modality approach. These clinical treatment failure rates remain high due to either intrinsic or extrinsic drug resistance. The chemoradioresistance in NPC is influenced by multiple critical factors including numerous proteins and molecules such as aberrant miRNAs, fusion genes, CSCs, BER pathways, etc. Accumulating evidence demonstrates a close association between chemotherapy radioresistance and these dysregulated gene expressions. An important signature in tumor progression and therapy is the advancement in genomic and molecular profiling. Microarray technology, which was developed utilizing high-throughput platforms during the last few decades, has proven to be a promising and efficient technique for detecting multiple significant cancer phenotypic features. Multitudes of proteins and biomarkers have been studied and these can be a remarkable avenue for developing a versatile and potent therapeutic agent in the near future. It is exquisitely vital to negate the chemoradioresistance efficiently, early in the disease process, in order to gain improved therapeutic outcomes. Hence, patient prognosis and quality of life can be dramatically improved.

## Figures and Tables

**Table 1:** Description profile of radioresistance mechanisms and their cellular pathway in NPC.

The Pathway or Gene Symbol	Description	Mechanism	References
Cancer Stem Cells (CSCs)	The expression of stem cell-related genes/proteins	Telomerase activity of NPC radioresistant cells	[39,45]
Epithelial–mesenchymal transition (*EMT*)	Cellular morphological changes	*EMT* gifts tumor cells with more powerful metastatic potency	[36,46]
Carnitine pal-mitoyl transferase 1 A (*CPT1A*) gene	Fatty acid oxidation (*FAO*) activity determination	*CPT1A-Rab14* interaction plays roles in *CPT1A*-mediated radiation resistance by facilitating fatty acid trafficking	[48]
*RARS-MAD1L1* Fusion Gene	Chromosomal aberration	Contributes to tumorigenesis and therapeutic resistance by inducing cancer stem cell (*CSC*)-like properties in NPC	[49,73]
Base Excision Repair (*BER*) Pathway	Single nucleotide polymorphisms (*SNPs)*	Mutation of *BER* pathway genes could increase the radiosensitivity by reducing the DNA repair ability	[41,42]
Endoplasmic reticulum (*ER*) stress pathway	Single nucleotide polymorphisms (*SNPs*)	Influences the binding ability of miRNAs and, consequently, the expression of target genes	[74]
*Wnt/β-catenin* pathway	Gene polymorphism	*β-catenin* may play an important oncogenic role in the migration and proliferation of NPC cells.	[39,58,75]
BST2 (*Tetherin, CD317*)	Upregulates anti-apoptotic genes (*Bcl-X_L_* and *livin*)	Knockdown of *BST2* in NPC cells sensitizes their response to cisplatin and promotes cisplatin-induced apoptosis	[54,57]
Long noncoding RNAs (*lncRNAs*)	Up/Downregulates the lncRNA. The long non-coding PTV1 induces radiosensitivity	Knockdown of PVT1 inhibits radioresistance by increasing cell apoptosis	[76,77]
Tyrosine-protein phosphatase (*SHP-1)*	Overexpression	Overexpressing *SHP-1* reverses the radiosensitive effect of quinalizarin	[78,79]
*c-Jun* gene	Downregulates *CNE-2R* cells due to G2/M phase arrest and enhanced cell apoptosis	Promotes tumor growth and progression, with *c-jun* gene binding to cyclin DI promoter	[80]
Micro RNA (miRNA)	MiR-19b-3p, miR-125b, miR-21, and miR-205 promote the radiotherapy resistance of nasopharyngeal carcinoma by regulating the *Bcl-2* gene family protein	miRNA is involved in the radiotherapy resistance of nasopharyngeal carcinoma by affecting apoptosis, DNA damage repair, and cell cycle progression of nasopharyngeal carcinoma cells	[27,62]

## Data Availability

No new data were created or analyzed in this study. All data included in this review have been previously published. If further specific data are needed, it may be provided by the corresponding author upon reasonable request.
